# CC-4066 therapy delivered to kidneys during cold storage and assessed with normothermic reperfusion is feasible and safe

**DOI:** 10.3389/frtra.2023.1166661

**Published:** 2023-05-11

**Authors:** Pommelien Meertens, Azita Mellati, Richard Dumbill, M. Letizia Lo Faro, Kaithlyn Rozenberg, John Mulvey, Hans Fliri, Rutger Ploeg, James Hunter

**Affiliations:** ^1^ Nuffield Department of Surgical Sciences, University of Oxford, Oxford, United Kingdoms; ^2^ Leiden University Medical Centre, Leiden University, Leiden, Netherlands; ^3^ Oxford University Hospital National Health Service (NHS) Foundation Trust, Oxford, United Kingdom; ^4^ Cypralis Ltd., Cambridge, United Kingdom; ^5^ University Hospitals of Coventry and Warwickshire National Health Service (NHS) Trust, Coventry, United Kingdom

**Keywords:** normothermic machine perfusion, CC4066, cyclophilin, inflammation, transplantation, ischemia-reperfusion injury, kidney

## Abstract

**Introduction:**

Currently there is an urgent need to translate interventions that may be beneficial to marginal donor kidneys prior to transplant, to improve their quality from bench to bedside. This project investigated the effects of CC-4066, a potent dual inhibitor of cyclophilin proteins A and D, treatment during static cold storage (SCS) in a porcine model of renal ischemia-reperfusion injury (IRI) using Normothermic Reperfusion (NR).

**Materials and methods:**

Porcine kidneys and autologous blood were retrieved in pairs from a local abattoir (*n* = 7). One kidney from each pair was randomly allocated to treatment and one allocated to control and flushed with preservation solution containing CC-4066 or vehicle. After 7 h of SCS kidneys underwent 3 h Normothermic Reperfusion (NR) with autologous whole blood while perfusion characteristics and samples were collected.

**Results:**

Perfusion and metabolic parameters showed similar trends and no statistical differences were observed between the groups. IL-6 showed a significant increase over time but no significant difference between groups (*p*-value 0.009 and 0.14 respectively, two-way ANOVA). Oxygen consumption and lactate levels were similar between groups but there was increased vacuolation on histology in the control group.

**Conclusions:**

The addition of CC-4066 during SCS of kidneys is safe and feasible and has no adverse or detrimental effects on perfusion during assessment on NR. There was no difference in cytokine levels although there was a trend towards less vacuolation on histology in the treatment group.

## 1. Introduction

Transplantation is the preferred treatment option for patients with end-stage-renal disease (ESRD) as it is more cost-effective, provides a better quality of life and improved survival compared with dialysis (1, 2). The high demand for donor organs and limited availability requires the use of older donors with co-morbidities referred to as extended criteria donors (ECD). Donors after circulatory death (DCD) undergo an inevitable period of warm ischemia which results in organ injury and a greater susceptibility to the subsequent ischemia-reperfusion injury (IRI) (3, 4). However, transplantation of ECD and DCD kidneys is still preferable compared to outcomes on continuous dialysis (5–8).

Marginal donor organs, including ECD and some DCD kidneys are more prone to IRI occurring after static cold storage (SCS) and transplantation in the recipient (9, 10). IRI is caused by the build-up of metabolites during anaerobic metabolism while the organ is preserved and by a cascading of effects modulated by these metabolites during reperfusion of the organ in the recipient (11–13). IRI can cause immune modulation, mitochondrial dysfunction, complement activation and oxidative damage to cells and tissues (14).

Mitochondria play a critical role in energy production and cell survival. They are particularly vulnerable to IRI due to the depletion of cellular ATP during ischemia and the subsequent generation of reactive oxygen species (ROS) during reperfusion. During ischemia, the depletion of cellular ATP and the accumulation of calcium ions lead to the opening of the mitochondrial permeability transition pore (mPTP), which further impairs ATP production and leads to mitochondrial dysfunction and consequently cellular oedema, rupture, and cell death induced by various pathways (14). Cyclophilins, in particular A and D are essential components of the mPTP and during IRI the opening of the mPTP facilitates the mitochondrial release of cytochrome c and calcium ions, causing cell damage and cell death. Upon reperfusion, the surge in ROS generation is also exacerbated by increased permeability of the mPTP. Therefore, targeting cyclophilins and blocking mPTP opening is a logical treatment strategy for ischemia-reperfusion injury (15).

The effects of IRI in kidneys from marginal donors are clinically represented by higher rates of delayed-graft function (DGF), defined as the need for dialysis in the first week post-transplantation, and primary non-function (PNF) of the donated kidney resulting in return to dialysis or re-transplantation for the recipient (9, 10).

A renewed interest in organ preservation has been triggered by the potential of using different strategies to improve quality of marginal grafts by reducing the impact of IRI (11). Normothermic Machine Perfusion (NMP) is a preservation method that utilizes extracorporeal membrane oxygenation and a blood-based perfusate solution. NMP provides a unique opportunity to deliver and assess the effects of therapies to an isolated organ and avoid most complications derived from systemic drug delivery (16). Normothermic Reperfusion (NR), which is NMP with whole blood, can be used to simulate ischemia-reperfusion injury conditions, as a surrogate for transplantation (17).

There are major challenges in drug development for ischemia-reperfusion injury and despite extensive research no new therapies have entered clinical practice in the last decades (18). CC-4066 (Cypralis, United Kingdom) is a potent dual inhibitor of Cyclophilin A and D proteins which may be beneficial in a broad range of diseases including IRI in kidney transplantation. Cyclophilin D inhibition prevents hypoxia-induced cell damage, while Cyclophilin A inhibition minimizes reperfusion injury (19–25).

This project aimed to assess the effect on kidney injury of CC-4066 delivered to injured pig kidneys during cold preservation, followed by Normothermic Reperfusion. More specifically, we investigated the effects of this therapeutic agent on inflammation and histology.

## 2. Material and methods

### 2.1. Study design

All experiments were performed using paired slaughterhouse pig kidneys obtained with autologous blood, from a local abattoir. This model was selected as it fulfils the 3Rs principles of animal research and the 15–20 min warm ischemia time simulates the injury sustained in human donation after circulatory death donor kidneys. Due to use of slaughterhouse kidneys in this study, no ethics committee approval was needed. Kidneys were retrieved as detailed below and flushed until the venous effluent was clear. Mini pig models were utilized in the development process of CC4066 to establish the efficacy to inhibit porcine (data not shown). Our preliminary proof of principle experiments had shown that CC-4066 added to Soltran™ (Baxter, UK) and delivered as a flush *via* the renal artery immediately following retrieval resulted in rapid intake and pharmacologically active tissue concentrations (Supplementary Figure A.1 and A.2., results not shown). At the time the experiments were performed Soltran™ (Baxter, UK) was the standard preservation fluid in the UK and was therefore selected (4). In addition, it was decided that it would be beneficial to expose the kidney to the drug as soon as possible and therefore the initial flush was performed with the same concentration of the compound.

**Figure 1:**
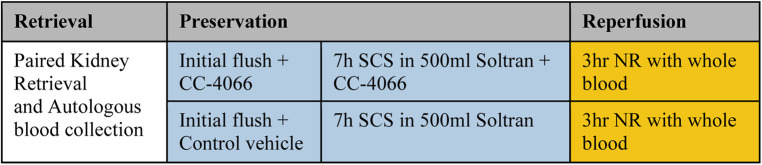
Overview perfusion experiments. SCS, static cold storage; NR, normothermic reperfusion.

Both kidneys were preserved in 500 ml Soltran™ (Baxter, UK) preservation fluid for 7 h during static cold storage, one with the addition of CC-4066 (*n* = 7), one with a control solution (*n* = 7) for 7 h, followed by 3 h of Normothermic Reperfusion (NR) with whole-blood perfusate (Figure 1). The outcomes investigated were: kidney perfusion characteristics, gas exchange, metabolic parameters and markers of function and injury as detailed below. Samples and data were collected during the reperfusion.

#### 2.1.1. Drug preparation

The flush and cold-storage solution were prepared as follows: 1 ml of 1 mmol/L stock solution of CC-4066 was added to 1l of Soltran to give a concentration of 15 µM. CC-4066 was dissolved in Kollisolv- PEG400, and the control group kidneys received the equivalent amount of Kollisolv PEG400 but without CC-4066. Once the kidneys were flushed, they were placed in an organ bag and put in an ice box and static cold stored (SCS) at <4°C for 7 h.

### 2.2. Retrieval and surgical procedures

Porcine kidneys were obtained from a local abattoir, as previously described (26). In brief, the animals were slaughtered and exsanguinated as per Home Office guidance under supervision of a veterinarian. Blood was collected in a (20,000 IU) heparinised (Fannin, Leopardstown, United Kingdom) container to prevent clotting and kidneys were immediately removed and flushed with preservation solution. Warm ischemia time was calculated from the time of exsanguination to cold flush. The kidneys were randomly assigned to either treatment or control group and were flushed with heparinised cooled preservation solution (Soltran, Baxter, UK), with either CC-4066 (treatment *n* = 7) or vehicle (control *n* = 7) until the effluent from the renal vein was clear. After retrieval and flush, the kidneys were placed in 500 ml of preservation solution (Soltran, Baxter, UK) containing either 15 µM CC-4066 or vehicle control (Kollisolv PEG400). The organ bags were placed in an ice box and static cold stored (SCS) at <4°C for 7 h. Kidneys were then transported back to the laboratory under these conditions.

### 2.3. Perfusate preparation for normothermic reperfusion

A whole-blood based perfusate was used as an experimental surrogate to simulate reperfusion in transplant surgery. The autologous whole blood collected at the abattoir was filtered using gauze within a funnel and was collected in a beaker until a volume of 1l was reached. This was divided equally between two perfusion circuits, reaching an amount of 500 ml whole blood in each reservoir. Supplements added included Amoxicillin-clavulanate (1,200 mg), Mannitol (10 mg), Creatinine (1000 µM), Insulin (5IU), Verapamil (0.75 mg). 5% Glucose and 10% calcium gluconate were added during NR to maintain physiological values according to Supplementary Table SB.1. A continuous infusion of verapamil of 0.25 mg/h was started after the perfusion commenced. During the perfusion blood-gas values were monitored and pH, glucose, Ca^2+^ were corrected to maintain physiological levels with the supplements described in Supplementary Table SB.2.

### 2.4. Perfusion circuits

**Figure 2:**
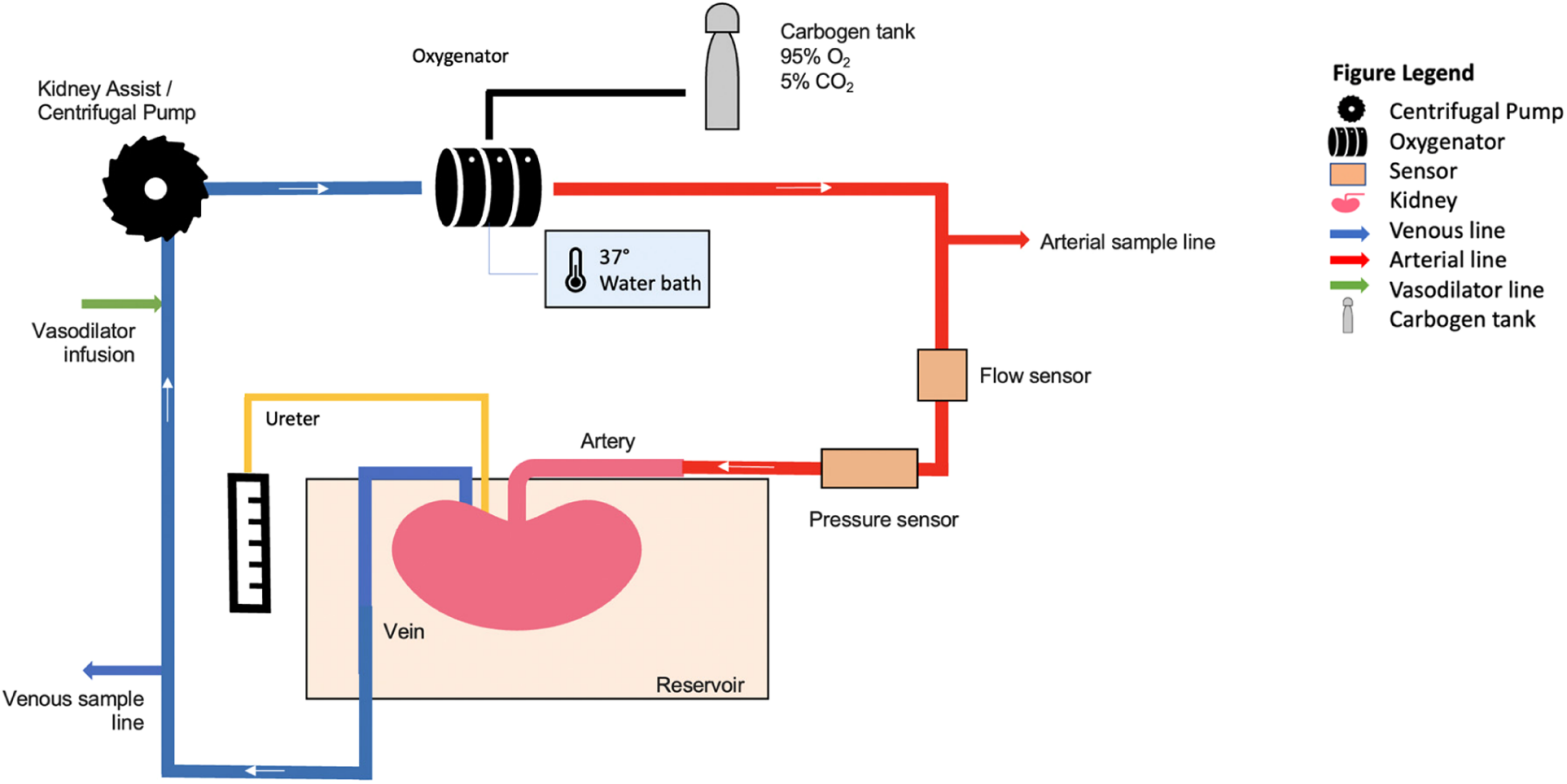
Schematic overview of the reperfusion circuit.

The NR system was based on the Kidney Assist (XVIVO, Groningen) device and consisted of a hollow fibre oxygenator (Hilte Lt 2500, Medos, Germany or Terumo FX05, Terumo Corp), a centrifugal pump (D3, Medos, Germany), medical-grade ¼ and 3/8 inch PVC and silicone tubing (ECC noDOP®, Raumedic, Germany) and an organ chamber. Figure 2 illustrates a schematic overview of the reperfusion circuit. Renal Blood Flow (RBF) was monitored using an ultrasonic clamp-on flow probe (Em-tec-Gmbh) and pressure was measured at the same height as the renal artery using a pressure transducer (Edwards Lifesciences). Temperature of the perfusate was kept at 37°C by using an oxygenator with a heat exchanger, connected to a water bath. Perfusion pressure was set at 70 mmHg as Mean Arterial Pressure (MAP). The ureter and renal artery were cannulated (Vycon, Ecouen, France and Infusion, Poland, respectively) and the kidney was perfused with whole blood for 3 h. The perfusate was oxygenated with 0.5 L/min carbogen (95% oxygen/5%CO2) (BOC Group, Guildford, United Kingdom).

During the perfusion, samples of urine, tissue and perfusate were collected. Urine sample aliquots were taken every 30 min and the remaining urine was recirculated to maintain the electrolyte balance and perfusate volume. Perfusate samples were taken at the start of the perfusion as baseline samples, at 90 min and at the end of the perfusion. Urine and perfusate samples were stored in ice and centrifuged at 18,000 × g for 12 min at 4°C, after which the supernatant was kept at −80°C for further analysis.

Blood-gas analyses of arterial and venous line samples were taken every 15 min for the first hour and every 30 min thereafter. Punch biopsies (4 mm, Stiefel, North Carolina, United States) from the upper pole of both kidneys were collected at three time points; start, middle and end of perfusion. Each biopsy was cut in half, with one half stored in 4% formalin for histology while the other was snap frozen in liquid nitrogen and later stored at −80°C for further analysis.

### 2.5. Arterial blood gas values and biochemistry analyses

Arterial blood gas and electrolytes were measured using the ABL90 FLEX blood gas analyser (Radiometer, Denmark).

### 2.6. Oxygen consumption

Renal oxygen consumption was calculated to estimate the metabolic activity of the kidney. This was done using the formula published by Venema et al. (5), which calculates the oxygen consumption in mL/min per 100 g kidney weight.

### 2.7. Cytokines

To assess inflammation, ELISA analyses for pro-inflammatory cytokines in plasma/perfusate samples were performed. TNF-α, IL-6 and IL-1β (porcine Quantikine Sandwich ELISA kit, R&D Systems Europe, Ltd. Abingdon, UK) were analysed in the perfusate samples according to manufacturer's instructions. An optimization experiment was performed for each analyte with pooled samples to ensure adequate recovery and to select the appropriate sample dilution. In summary, the reagents, assay buffer and standards were prepared after which the perfusate samples, spiked samples, standards and positive controls were added to the wells of the ELISA plate. For the IL-1β and TNF-α the plate was pre-coated with specific (IL-1β and TNF-α) antibodies, for the IL-6 the plate was coated and prepared the day before. After an incubation period of 2 h, unbound material was washed away and the detection antibody was added to bind to the captured analyte. After another 2 h of incubation the plate was washed 4× times. The last step was the addition of Tetramethylbenzidine and hydrogen peroxide (to allow for colour development, as product of the reaction) and incubation for another 30 min in the dark, after which the stop solution was added and the plate was analysed at 450 and 560 nm on a microplate reader (iMark Bio-Rad). The levels of the cytokines were calculated by interpolation from the standard curve.

### 2.8. Histology

The biopsies were fixed in formalin, at time of collection, and then were subsequently fixed and embedded in paraffin blocks. 4 µm-thick slides were cut and stained with Hematoxylin and Eosin (H&E) (Leica ST infinity stain, Leica Biosystems, Milton Keynes, UK) following manufacturer's instructions. The biopsies were reviewed by a consultant renal pathologist and scored blindly using an injury severity score that combined: tubular dilation, interstitial edema, tubular casts and tubular vacuolation (27). For each parameter, a percentage was assessed and then converted in 0–3 scores as follows: 0 = 0%–1%; 1 = 1%–10%; 2 = 10%–25%; 3 = >25%.

### 2.9. Statistical analyses

Statistical analysis was performed using GraphPad Prism 9.0. For normality testing the Shapiro-Wilko test was used. Descriptive characteristics as WIT, CIT and weight before and after NR were analysed with unpaired *t*-test. For variables continuously monitored during perfusion (renal blood flow RBF, Intra-renal resistance IRR and Lactate), the Area Under the Curve (AUC) was calculated before any further statistical analysis. The AUC of the two groups (CC-4066 vs. Control) was then compared with an unpaired *t*-test. For metabolic parameters, the values were analysed using a two-way ANOVA with mixed effects model to explore the effect over time and between the groups, referred to as repeated measures in the text. If the data did not pass the normality test, the data were log-transformed to achieve normality. A *p*-value of <0.05 was considered significant. For the cytokine analysis a two-way ANOVA with mixed effects model was used. For categorical variables, such as histology scores, chi-squared or Fisher's exact test were used.

## 3. Results

### 3.1. Machine perfusion characteristics and haemodynamics

The total WIT (Warm Ischemic Time) (mean ± SD) was the same for the two groups as the kidneys were retrieved as a pair. There was no statistical difference between the CIT (Cold Ischemic Time) or kidney weight in both groups (Table 1).

**Table 1:** Descriptive statistics of warm and cold ischemic times and kidney weight.

	Control (*n* = 6)	CC-4066 (*n* = 5)	*p*-value
Warm Ischemic Time (WIT)	15 ± 1		
Cold Ischemic Time (CIT) (hr:min ± min)	07:28 ± 29	07:29 ± 37	Ns, 0.97
Weight before NR (g)	187 ± 52	182 ± 58	Ns, 0.88
Weight after NR (g)	215 ± 65	211 ± 67	Ns, 0.91
% Weight gain	15 ± 11	18 ± 7	Ns, 0.79
Mean ± SD, unpaired t-test	* *	* *	* *

A total of 7 paired kidney perfusions were conducted according to the protocol described above. Three kidneys were excluded due to technical problems during the experiments which resulted in *n* = 6 kidneys in the control group, and *n* = 5 kidneys in the treatment group (CC-4066).

**Figure 3:**
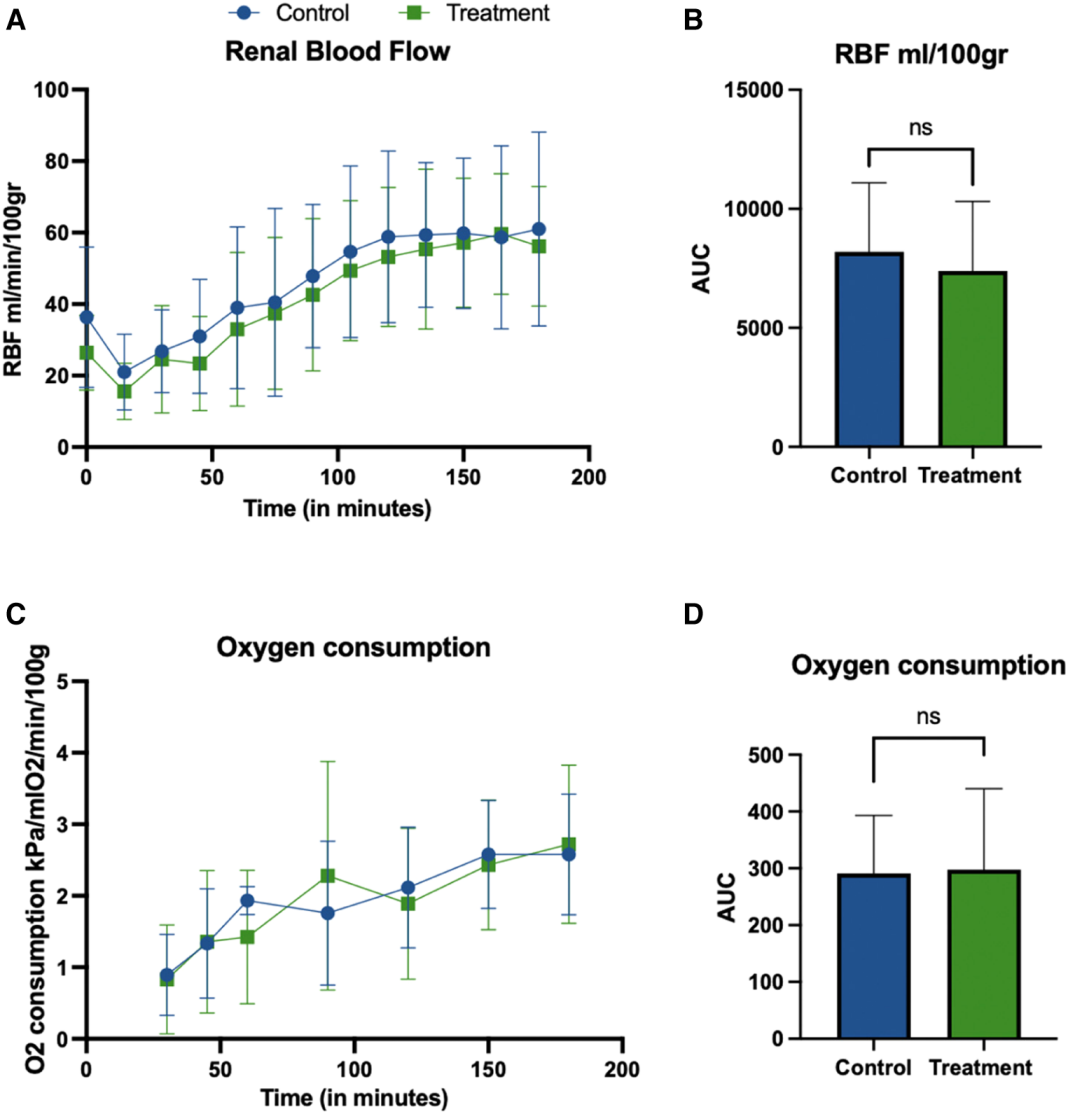
Renal blood flow (ml/min/100 gr) (**A,B**) during NR. The perfusion flow (**A**) shows the same trend in both groups, with initial dip and increase after 60 min of perfusion with no statistical difference between the groups. Graphs report mean ± SD of individual time points (**A**) and AUC (**B**). There was no significant difference between the groups in flow AUC (**B**) (*p*-value 0.66, unpaired *t*-test). Oxygen consumption during NR (**C,D**). Graphs show mean ± SD of Oxygen consumption calculated as described in section 2.6. No statistical differences between the groups were observed for the AUC (*p* = 0.9295, unpaired *t*-test).

The MAP was kept at 70 mmHg during the perfusion in both groups. Figures 3A,B show the RBF (ml/min/100 g) over time (min) in the control and treatment group. Similar flow rates were seen in both groups at the end of NR (55 ± 25 ml/min/100 g vs. 53 ± 17 ml/min/100 g). There was no statistical difference between the groups for the flow AUCs (*p* 0.66, unpaired *t*-test), Figure 3B. Resistance showed an initial peak with a maximum mean resistance of 2.61 ± 1.49 in the control group, vs. 1.81 ± 0.15 in the treatment group. At the end of perfusion, the mean resistance was 1.21 ± 1.24 in the control group vs. 0.94 ± 0.34 in the treatment group. There was no significant difference between the groups in the resistance AUC (*p*-value 0.35, unpaired *t*-test) (data not shown).

The mean cumulative urine production in the control group over 3 h of reperfusion was 9 ± 12 ml/100 gr vs. 7 ± 8 ml/100 gr in the treatment group (*p* = ns).

### 3.2. Metabolic parameters

Most metabolic parameters, including pCO_2_, hematocrit, sodium and calcium were stable during perfusion and no statistical differences were observed between groups. No significant differences between any of the parameters were detected. The pH and vHCO_3_ increased significantly over time but were not different between the groups (pH, *p*-value: time 0.0025, treatment 0.5312; vHCO_3_, *p*-value time 0.0031, treatment 0.6370, two-way ANOVA with repeated measures). The NR metabolic parameters, for the start and end of reperfusion are shown in Table 2 as mean ± standard deviation (SD).

**Table 2:** Metabolic parameters and electrolytes during perfusion.

Mean** **±** **SD	** **	Control (*n* =** **6)	Treatment (*n* =** **5)	*p*-value
pH	Start	7.42** **±** **0.08	7.45** **±** **0.10	Time: 0.0025* Treatment: 0.53
	End	7.46** **±** **0.13	7.48** **±** **0.09	
paO_2_ (kPa)	Start	79.9** **±** **4.8	79.4** **±** **3.7	Time: 0.57 Treatment: 0.43
	End	78.8** **±** **3.4	77.3** **±** **12.1	
pCO_2_ (kPa)	Start	4.4** **±** **0.9	4.3** **±** **0.5	Time 0.39 Treatment 0.78
	End	4.7** **±** **0.4	4.7** **±** **0.4	
pvO_2_ (kPa)	Start	6.7** **±** **1.5	5.8** **±** **1.2	Time: 0.53 Treatment: 0.53
	End	7.0** **±** **1.2	6.4** **±** **0.6	
aHCO_3_ (mmol/L)	Start	23.1** **±** **3.3	23.5** **±** **4.2	Time: 0.31 Treatment: 0.59
	End	24.9** **±** **10.1	27.8** **±** **3.8	
Hematocrit (%)	Start	36.7** **±** **5.7	38.2** **±** **5.9	Time: 0.55 Treatment: 0.59
	End	37.0** **±** **3.6	38.8** **±** **6.1	
vHCO_3_ (mmol/L)	Start	19.9** **±** **2.7	20.4** **±** **5.8	Time: 0.0031* Treatment: 0.64
	End	26.6** **±** **6.1	28.0** **±** **3.7	
Glucose (mmol/L)	Start	5.1** **±** **1.3	4.9** **±** **1.1	Time: 0.76 Treatment: 0.37
	End	4.5** **±** **0.9	6.1** **±** **4.2	
Sodium (mmol/L)	Start	136.8** **±** **6.1	138.4** **±** **7.3	Time: 0.13 Treatment: 0.91
	End	142.2** **±** **3.3	140.0** **±** **8.0	
Potassium (mmol/L)	Start	13.2** **±** **3.7	13.0** **±** **3.7	Time: 0.17 Treatment: 0.99
	End	11.3** **±** **1.3	10.8** **±** **2.1	
Calcium (mmol/L)	Start	0.72** **±** **0.17	0.80** **±** **0.27	Time: 0.18 Treatment: 0.47
	End	1.02** **±** **0.17	1.15** **±** **0.20	

* Indicates significant *p*-value.

The mean ± SD for perfusate Lactate at the start of perfusion was 4.6 ± 2.1 mmol/L in the control groups vs. 5.0 ± 1.8 mmol/L in the treatment group. At the end of the perfusion these were 5.7 ± 1.1 vs. 6.1 ± 1.7, respectively. No statistical differences were observed between the groups for Lactate AUC (*p*-value 0.61, unpaired *t*-test) (Supplementary Figure SC.1).

Oxygen consumption was calculated during NR and shown in Figures 3C,D and was calculated as described in section 2.6.6. The mean ± SD for oxygen consumption at the start of perfusion was 0.90 ± 0.57 kPa/mlO_2_/min/100 gr in the control groups vs. 0.83 ± 0.76 kPa/mlO_2_/min/100 gr in the treatment group. At the end of the perfusion these were 2.58 ± 0.84 vs. 2.72 ± 1.10, respectively. No statistical differences groups were observed for the AUC (*p* = 0.9295, unpaired *t*-test).

### 3.3. Cytokine analysis

**Figure 4:**
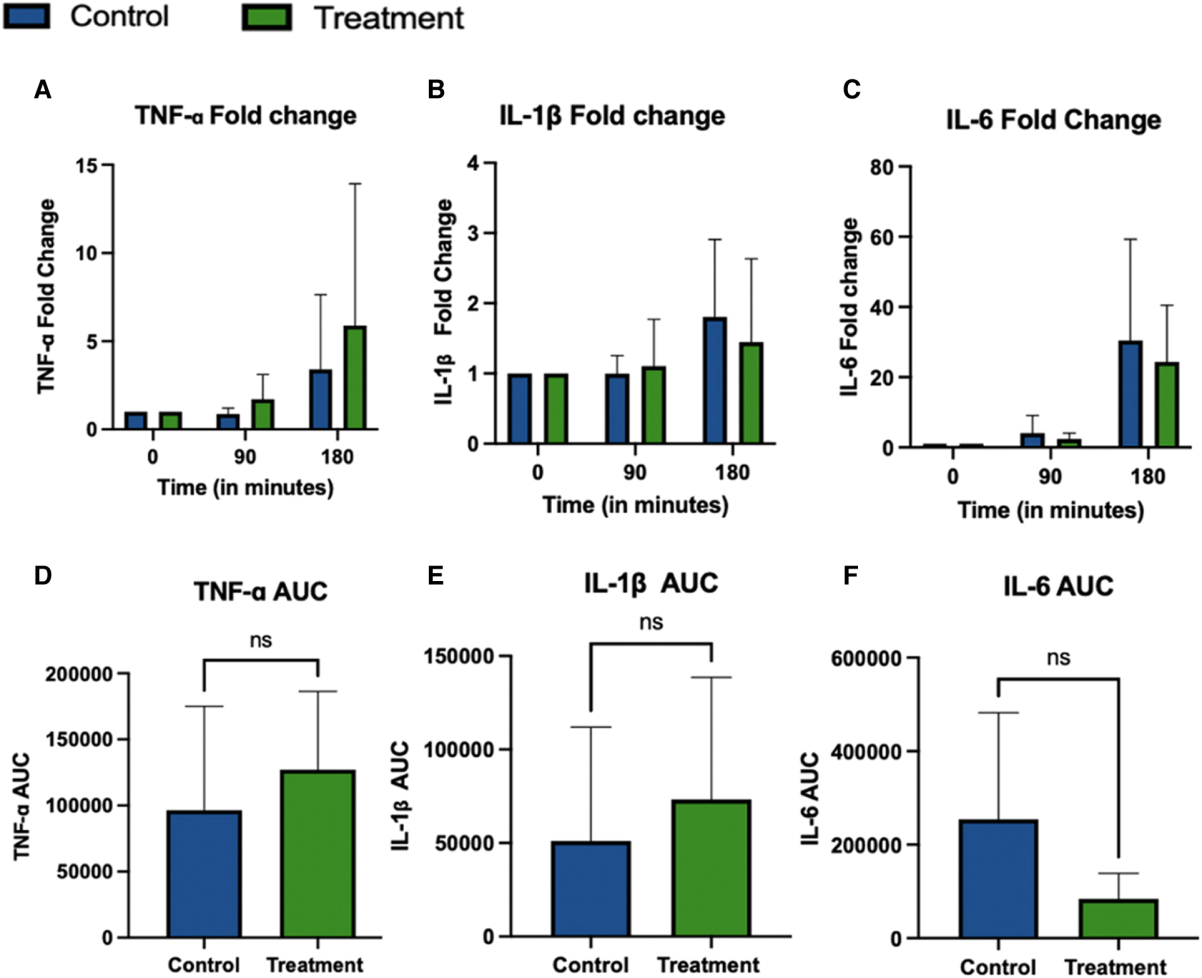
TNF-α, IL-1β and IL-6 cytokine concentrations in perfusate during 3 h NR. Due to out-of-range levels in one pair, 2 kidneys were excluded from TNF-α analysis, CC-4066 (*n* = 4) and control (*n* = 5). For all other cytokines *n* = 5 in Treatment and *n* = 6 in the control group were included for analysis. Graphs represents mean ± SD of the fold change (**A–C**) and AUC **(D–F**).

Results for the TNF-α ELISA analysis are shown in Figures 4A,D, with (A) presenting fold change from baseline (*t* = 0 min) and D representing TNF-α AUC. No statistical differences in perfusate TNF-α levels were observed over time or between the two groups (*p*-value 0.13 and 0.43, two-way ANOVA). Similarly, fold change (*p*-value 0.06 and 0.46, two-way ANOVA) and AUC (*p*-value 0.53, unpaired *t*-test) did not show a significant difference. Due to out-of-range levels in one pair, 2 kidneys were excluded from TNF-α analysis, CC-4066 (*n* = 4) and Control (*n* = 5).

IL-1β concentrations are shown in Figures 4B,E show no statistical difference over time or between groups (*p*-value 0.43 and 0.59, two-way ANOVA). IL-1β Fold change does show a significant increase over time, but no difference was detected between groups (*p*-value 0.03 and 0.79, respectively; two-way ANOVA). IL-1β AUC also showed no statistical difference between the groups (*p*-value 0.57, unpaired *t*-test). Graphs in Figures 4C,F show the fold change and AUC of perfusate IL-6 during perfusion. There was a statistically significant increase of IL-6 over time for the absolute values, but no statistical difference between groups (*p*-values 0.009 and 0.14 respectively, two-way ANOVA with repeated measures). Similarly, the fold change showed a significant difference over time but not between the groups, (*p*-value 0.004 and 0.62 two-way ANOVA with repeated measures), the fold change showed a significant difference over time but not between the groups, (*p*-value 0.004 and 0.62 two-way ANOVA with repeated measures) and the AUC also showed no statistical difference between the groups (*p*-value 0.14, unpaired *t*-test).

### 3.4. Histology

**Figure 5:**
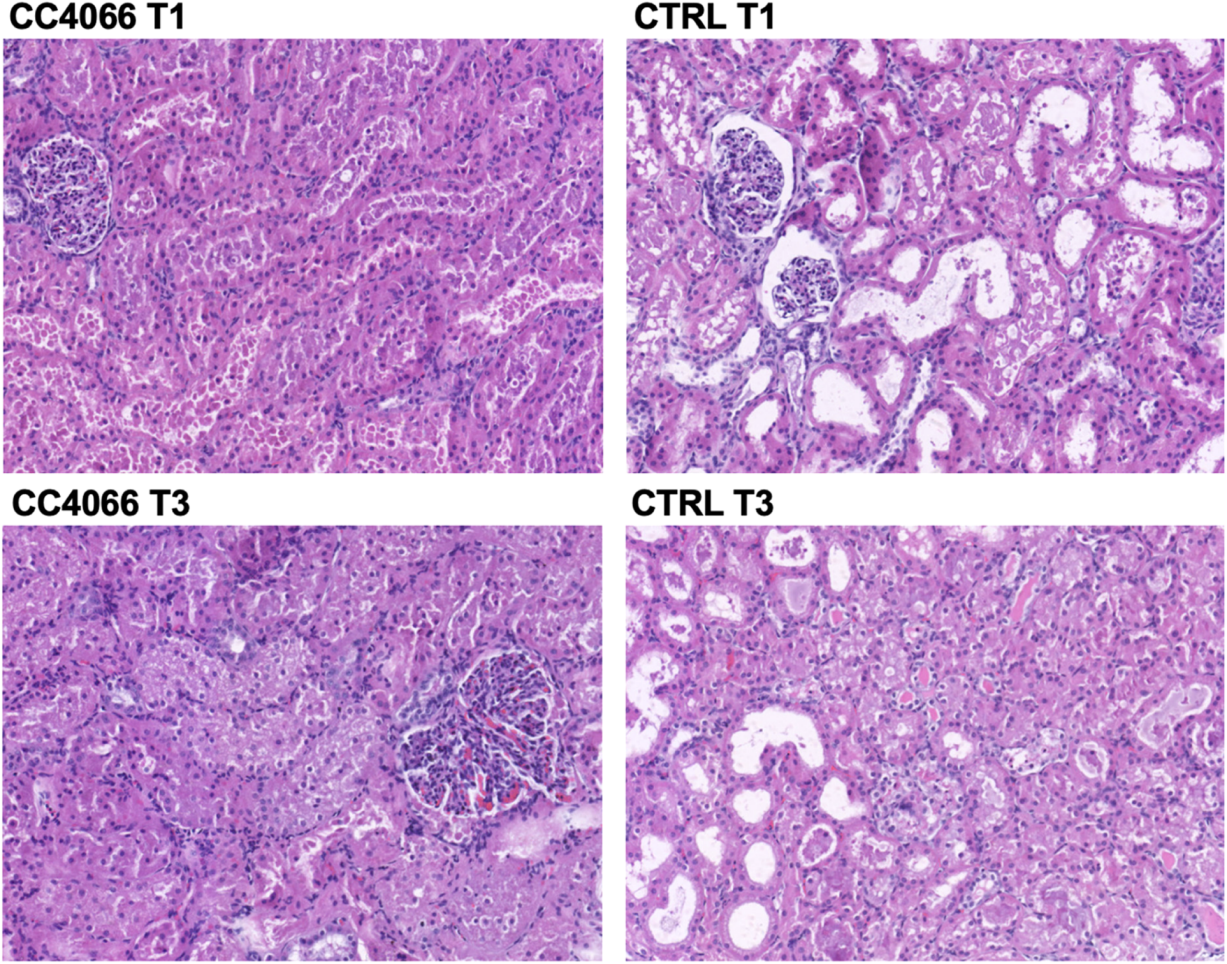
Representative pathology slides from one pair of kidneys from the same pig; control (CTRL) and treatment (CC4066) at the end of cold storage and before start of perfusion (T1) and the end of 3 h reperfusion (T3). Increased vacuolation can be observed in the control group images.

The percentage scores that are displayed in Table 3. This analysis represents categorical variables and as per the statistical section in the methods, Fisher's exact and Chi-squared tests were used for analysis. However, due to the small numbers and absence of data in some of the percentage categories it was not possible to perform those tests. Data from Table 3 suggest that there was more severe vacuolation in the control group and appreciating the limitations of the statistical tests we performed further analysis. This showed no difference in percentage (%) vacuolation between the CC-4066 and control groups at T0 (start of cold storage, unpaired t test, *p* 0.98) or T1 (before the start of NR, unpaired t test, *p* 0.14). There was an increase in % vacuolation from T1 to T3 (after 3 h of NR) in the treatment and control groups (*p* 0.03 and *p* 0.002 unpaired t test, respectively). Percentage vacuolation was also significantly higher in the control group at T3 compared with the treatment group at T3 (unpaired t test, *p* 0.03), with pathology slides shown in Figure 5. No differences were observed between groups or between time points for tubular dilation, acute tubular injury score or % oedema and % red cell casts.

**Table 3:** Percentage tubular dilation (TD) and vaculation (V).

%	Control T1		Treatment T1		Control T3		Treatment T3	
	TD	V	TD	V	TD	V	TD	V
<30	1	5	1	5	2	0	2	3
30–60	1	0	2	0	1	4	1	2
>60	3	0	2	0	2	1	2	0

This table illustrates the percentage tubular dilatation (TD) and vacuolation (V) on histological scoring for kidneys in control and treatment groups at T1 (before the start of reperfusion) and T3 (following 3** **h reperfusion). Scores are grouped as <30%, 30–60% and >60% and the values represent the number of kidneys in each group with that score.

## 4. Discussion

Improving the quality of donor organs during preservation could play an important role in addressing the issue of donor kidney shortage and is therefore relevant. Treatments that reduce ischemia-reperfusion injury (IRI) will be a part of the solution to maintain or improve the quality of the kidney during preservation. Our preliminary studies showed that delivering CC-4066 during the initial flush resulted in rapid uptake and pharmacologically relevant tissue concentrations at the end of 7 h cold storage. The aim of this study was to investigate whether the addition of CC-4066, an inhibitor of cyclophilins A and D, during preservation would reduce the impact of IRI. A porcine model was used with 7 h SCS followed by 3-hour Normothermic Reperfusion (NR) as a pre-clinical research tool. The main finding of this study was that adding CC-4066 during SCS, followed by NR was safe and feasible and had no adverse effects on kidney perfusion.

This study did not find a significant impact of the compound CC-4066 on perfusion parameters of renal blood flow or resistance during perfusion. We did not observe a statistical difference between the two groups, and it was clear that addition of CC-4066 during SCS did not adversely impact perfusion parameters. Following the initiation of perfusion there was a dip in RBF and concomitant increase in IRR, which resolved after about 30 min. This phenomenon has been observed by others and is likely to be due to the resolution of vasoconstriction on reperfusion (28). Urine production varied greatly between individual pigs, as has also been observed in other studies working with porcine abattoir kidneys (29).

Glucose consumption by the kidney during perfusion can be considered as an indicator of metabolic activity of the organ, and no difference was observed between the groups. Lactate, a byproduct of anaerobic respiration, has been shown to increase during perfusion before reaching a plateau or decreasing (30). We observed a similar trend, with an initial peak corresponding with lower flows and higher resistance after which a trend towards stabilization of the perfusate lactate levels was observed with no significant difference between the groups. Interpretation of lactate levels remains challenging as its implications during NMP or NR are unclear. Previous work has shown that kidneys with increasing lactate in the context of low oxygen consumption and acidosis during NMP had lower blood flow and worse perfusion parameters (31, 32).

In our study the treatment of kidneys during SCS followed by normothermic reperfusion with whole blood was performed to mimic IRI conditions and assess the effects of the drug. We have previously shown that drug delivery during SCS followed by NR can be used as a means of assessment (17, 33) This is different from previous studies which have used NMP and NR as an administration platform for different compounds targeting ischemia-reperfusion injury. Administration during SCS has a clear advantage over administration during NMP/NR as it can be implemented clinically. One study administering an anti-CD47 blocking antibody during NMP in a porcine model found improved RBF, reduced IRR, reduction of oxidative stress and histological differences when compared to the control group, however tubular and glomerular function were not influenced by the treatment (34). However, as healthy pig kidneys were perfused with leukocyte-depleted blood for 1 h, and different drug administration routes were applied, these results are not directly comparable but certainly support the usefulness of the NMP and NR platform for delivering and testing different types of compounds.

The reperfusion phase in this study is designed to be an experimental surrogate, simulating IRI during transplantation. We acknowledge that there are limitations with this model and that potentially using allogeneic or xeno blood would approach transplant conditions even more instead of autologous blood. However, blood matching and the facility of a “bank pig blood” were not available at the time of performing the research. This model is the best representation available to our group at the time and “whole-blood” included leucocytes, complement and plasma proteins to simulate reperfusion. Therefore mechanisms that occur during IRI such as the generation of Reactive Oxygen Species (ROS) and activation of endothelial and immune cells to produce pro-inflammatory cytokines can be assessed using our model. The cytokines analysed were selected from a large repertoire based on their mechanisms of action and previous studies using CC-4066 and similar cyclophilin compounds.

TNF-α, a cytokine mainly produced by macrophages in the presence of ROS, plays a central role in apoptosis and inflammation due to cell activation and cell-cell recruitment. It is rapidly released after trauma and infection and considered as an early mediator of inflamed tissue (35–38). IL-1β is also a key mediator of the inflammatory response, and has been associated with kidney injury (39). IL-1β is produced in response to DAMPs (damage-associated molecular patterns), like Cyclophilin A, its levels were an interesting endpoint for our analysis. IL-1β and TNF-α also influence the production of IL-6 *via* transcription factors. IL-6 is produced in response to IRI, has a lot of different functions in different cell types (hepatocytes, bone marrow, CD4 and CD8 cells and B-cells) and is up-regulated very early during IRI, which makes it an ideal marker in this study.

There were no significant differences in levels of TNF-α, IL-6 or IL1β. There was a large variation in IL-6 levels in the control group and with greater sample numbers there may have been a difference between the groups. Previous work has demonstrated that CC-4066 does inhibit IL-6 production (unpublished data). Other studies investigating cytokine levels during NMP/NR showed comparable results to ours (40–42). The 3 h period of reperfusion may not be long enough to observe a change in all cytokine levels and a longer duration would be beneficial in future experiments.

Histological assessment showed that there was a difference in vacuolation between baseline and end of perfusion in both groups and there was a less vacuolation in the treatment group at the end of perfusion. All other histological analyses did not show differences between groups, in particular there was no evidence of drug toxicity in the treatment group. Previous work has shown that histological changes, including vacuolation, occur during NMP even in uninjured kidneys (32). The reason for this is unclear but it may be that CC-4066 is protective against IRI-induced vacuolation given the absence of any osmotic difference between the groups and no other chemical toxicity.

One of the limitations of the study is the variability observed between the different abattoir pigs, which makes detecting subtle, early differences between the groups more challenging. However, the use of such animals is ethical, in line with sustainability and the principles of the 3Rs, and we have shown that despite the variation can be used reproducibly(17). Humans also have similar anatomy and physiology to pigs and studies have shown that their reaction to ischemic injury also shares similarities with human kidney injury response. Given that 3 kidneys were excluded from some of the analyses, the inclusion of more kidney pairs would be beneficial in future. In addition, the period of 3 h of NR might have been too short to observe molecular changes at tissue level and a longer period might be more effective. The magnitude of warm ischemic injury of this model was reflected in the modest renal blood flow values and this may limit oxygen consumption and result in ongoing warm ischemia.

In conclusion, this study showed that the addition of CC-4066 to kidneys during static cold storage was safe and feasible and had no adverse effects on the kidneys during assessment by Normothermic Reperfusion. Levels of tissue injury markers and cytokines in the perfusate showed no difference between the groups for TNF-α, IL-1β and IL-6, however there was a trend towards less vacuolation on histology in the CC-4066 treated group. The findings in this pre-clinical study support future research to get a more detailed insight about the additional biological effects of treatment with this promising compound.

## Data Availability

The raw data supporting the conclusions of this article will be made available by the authors, without undue reservation.

## Supplementary A.1

Proof-of-principle experiments.

## Supplementary Table A.1

Overview proof-of-principle experiments.

## Supplementary Table B.1

Whole blood perfusate composition.

## Supplementary Table B.2

Pig NMP physiological blood gas and electrolytes values.

## Supplementary Figure C.1

Lactate concentration during Normothermic Reperfusion. Lactate concentration during NR. Pig kidneys were retrieved, cold stored with treatment of CC-4066 (n=5) or Control (n=6) and then reperfused for 180 minutes on NR at MAP of 70mmHg and temperature of 37°C. Graphs show mean ± SD of perfusate lactate levels. Both groups show same trend with increasing levels at the beginning and plateauing during perfusion. No statistical differences between the groups were observed (p-value 0.6069, unpaired t-test).
