# Supplementary material for: CC-4066 therapy delivered to kidneys during cold storage and assessed with normothermic reperfusion is feasible and safe
Source: Front Transplant. 2023 May 11;2:1166661. doi: 10.3389/frtra.2023.1166661 (PMC11270626; doi:10.3389/frtra.2023.1166661)
Supplement: Supplementary file 1 [file Datasheet1.pdf]

Supplementary Material

CC-4066 therapy delivered to kidneys during cold storage and assessed with normothermic reperfusion is feasible and safe.

Pommelien Meertens[1,2], Azita Mellati[1], Richard Dumbill[1,3] , M. Letizia Lo Faro[1], Kaithlyn Rozenberg[1], John Mulvey[1], Hans Fliri[4] , Rutger Ploeg[1,2,3], James Hunter[1,5]

1. Nuffield Department of Surgical Sciences, University of Oxford, United Kingdom
2. Leiden University Medical Centre, Leiden University, Leiden, Netherlands.
3. Oxford University Hospital National Health Service (NHS) Foundation Trust, Oxford, United Kingdom.
4. Cypralis Ltd, Cambridge, United Kingdom
5. University Hospitals of Coventry and Warwickshire National Health Service (NHS) Trust, Coventry, United Kingdom.

*** Correspondence:**
Pommelien Meertens
pommelien.meertens@nds.ox.ac.uk
James Hunter
james.hunter@nds.ox.ac.uk

1 Supplementary Data

A.1 Proof-of-Principle Experiments

To ensure adequate delivery of CC-4066, stability in the preservation solution and to exclude the possibility of interaction with the plastic tubing, plastic bags and plastic components of the circuit, preliminary experiments were performed. Soltran (Baxter, UK) and UW® (Bridge to Life, Il,US), the two most frequently used preservation solutions, used in the UK for human kidney retrieval were tested.

Two pairs of kidneys were retrieved from the abattoir. After standard retrieval protocol the kidneys were flushed with either Soltran or UW until the venous effluent became clear and placed in an organ bag. 500ml of 15 micromolar (µM) CC-4066-treated preservation solution were prepared, as described above. The kidney was submerged in 400ml of the solution and the remaining 100ml was flushed into the kidney. The control kidney was submerged in 500ml untreated preservation solution.

During these experiments perfusion fluid samples and biopsies were taken from the upper pole, lower pole, midpolar region, anterior and posterior kidney surface. These samples were sent to the Cypralis laboratory for assessment.

After analysis of these samples both preservation solutions demonstrated adequate delivery of CC-4066 to the kidneys. Since Soltran was the standard preservation fluid in the UK it was decided to use this in the main experiments(4). In addition, it was decided that it would be beneficial to expose the kidney to the drug as soon as possible and therefore the initial flush was performed with the same concentration of the compound.

Table A.1: Overview Proof-of-Principle Experiments

Retrieval	Preservation	
Paired Kidney Retrieval	Initial flush	8h SCS in 500ml Soltran/UW with 15µM CC-4066
	Initial flush	8h SCS in 500ml Soltran/UW

Table B.1: Whole blood perfusate composition

Whole blood perfusate		
**Ingredient**	**Amount**	**Company**
Whole blood	500 ml	NA
Amoxicillin-clavulanate	1200 mg	Bowmed, UK
Mannitol	10 mg	Sigma Aldrich, UK
Creatinine	1000 µmol	Sigma Aldrich, UK
Insulin	5IU	Novo, nordisk, UK
**Continous administration**		
Verapamil	0.25mg/h	Mylan products, UK
**Other supplements (added if needed)**		
8.4% sodium bicarbonate		B. Braun, Germany
5% Glucose		B. Braun, Germany
10% calcium gluconate		Hameln pharmaceuticals, UK

Table B.2: NMP Physiological blood gas and electrolytes values

Blood gas values and electrolytes	Pig NMP values	Normal Pig values	Human values NHS 2017	Unit
pH	7.35 - 7.50	7.35 – 7.50	7.35 – 7.45	
Partial pressure carbon dioxide (pCO_2)	4 - 6	4 – 6	4 – 6	kPa
Partial pressure oxygen (pO_2)	>60	10 – 12	10 – 12	kPa
Oxygen saturation (sO_2)	100	100	100	%
Haematocrit (Hct)	25-30	24 – 33	40 - 50	%
Sodium (Na^+)	129 - 145	129 – 145	133 - 146	mmol/L
Potassium (K^+)	3.5 – 5.3	3.5 – 5.3	3.5 – 5.3	mmol/L
Ionized Calcium (iCa^{2+})	0.9 – 1.1	0.9 – 1.1	1.2 - 1.3	mmol/L
Glucose	4.0 – 9.0	2.6 – 6.5	5.5 – 6.9	mmol/L
Lactate	0.0 – 2.5	0.5 – 2.5	0.5 – 2.0	mmol/L
Bicarbonate (HCO_3^-)	22 – 33	22 – 33	22 – 29	mmol/L
Addition of additives:<br>Ionised calcium range 0.9 – 1.1 mmol/L<br>If iCa^{2+} is between 0.8 and 0.9 → add 1ml of calcium gluconate<br>If iCa^{2+} is below 0.8 → add 2 ml of calcium gluconate				

Figure C.1: Lactate concentration during Normothermic Reperfusion

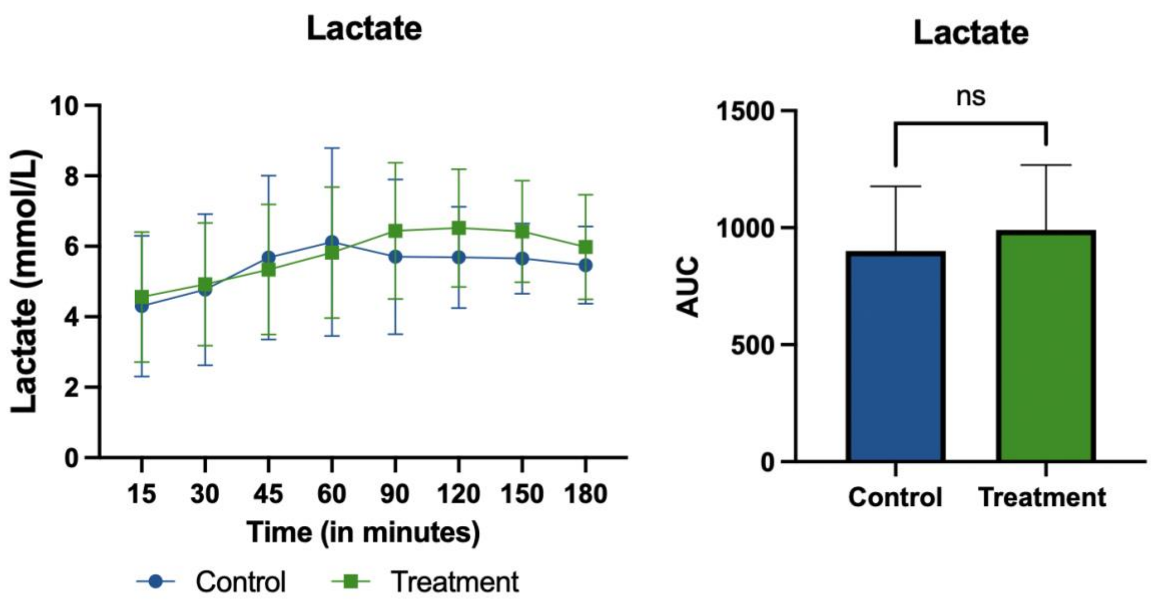

Figure C.1: Lactate concentration during NR. Pig kidneys were retrieved, cold stored with treatment of CC-4066 (n=5) or Control (n=6) and then reperfused for 180 minutes on NR at MAP of 70mmHg and temperature of 37°C. Graphs show mean ± SD of perfusate lactate levels. Both groups show same trend with increasing levels at the beginning and plateauing during perfusion. No statistical differences between the groups were observed (p-value 0.6069, unpaired t-test).
